# Air pollution and hemorrhagic fever with renal syndrome in South Korea: an ecological correlation study

**DOI:** 10.1186/1471-2458-13-347

**Published:** 2013-04-15

**Authors:** Seung Seok Han, Sunhee Kim, Yunhee Choi, Suhnggwon Kim, Yon Su Kim

**Affiliations:** 1 Department of Internal Medicine, Seoul National University College of Medicine, 101 Daehakro, Jongno-gu, Seoul 110-744, Korea; 2 Medical Research Collaborating Center, Seoul National University College of Medicine, Seoul, Korea; 3 Kidney Research Institute, Seoul National University, Seoul, Korea

**Keywords:** Air pollution, Hantavirus, Hemorrhagic fever with renal syndrome, Particulate matter, Infection

## Abstract

**Background:**

The effects of air pollution on the respiratory and cardiovascular systems, and the resulting impacts on public health, have been widely studied. However, little is known about the effect of air pollution on the occurrence of hemorrhagic fever with renal syndrome (HFRS), a rodent-borne infectious disease. In this study, we evaluated the correlation between air pollution and HFRS incidence from 2001 to 2010, and estimated the significance of the correlation under the effect of climate variables.

**Methods:**

We obtained data regarding HFRS, particulate matter smaller than 10 μm (PM_10_) as an index of air pollution, and climate variables including temperature, humidity, and precipitation from the national database of South Korea. Poisson regression models were established to predict the number of HFRS cases using air pollution and climate variables with different time lags. We then compared the ability of the climate model and the combined climate and air pollution model to predict the occurrence of HFRS.

**Results:**

The correlations between PM_10_ and HFRS were significant in univariate analyses, although the direction of the correlations changed according to the time lags. In multivariate analyses of adjusted climate variables, the effects of PM_10_ with time lags were different. However, PM_10_ without time lags was selected in the final model for predicting HFRS cases. The model that combined climate and PM_10_ data was a better predictor of HFRS cases than the model that used only climate data, for both the study period and the year 2011.

**Conclusions:**

This is the first report to document an association between HFRS and PM_10_ level.

## Background

Hemorrhagic fever with renal syndrome (HFRS) is caused by a group of viruses that show similar clinical manifestations [1,2]. The etiologic agent is a hantavirus belonging to the *Bunyaviridae* family [3]. Rodents are the predominant reservoir of hantavirus and excrete virus-containing urine, feces, and saliva when chronically infected. Hantaviruses are then transmitted to humans mainly via aerosols generated from animal secretions [4]. HFRS cases have been reported throughout the world, although more than 90% of cases occur in Asian countries, including China, Japan, and Korea. In North and South America, hantavirus causes another form of febrile illness, hantavirus pulmonary syndrome [5]. The first documented HFRS outbreak occurred during the Korean War, when more than 3200 soldiers were infected and approximately 400 patients died [6]. The hantavirus was first isolated from rodents in 1978 and named after the Hantan River area in South Korea (Hantaan virus) by Ho Wang Lee [7]. In 1982, researchers identified the Seoul virus, the only hantavirus known to have spread worldwide [8]. Although the cause and transmission of HFRS have been extensively investigated, the incidence of HFRS has not decreased, and more than 300 cases of HFRS develop annually in South Korea. The reason for the continued occurrence of HFRS, despite intensified sanitation and preventive actions, is not well understood.

It is reasonable to assume that climate change affects the incidence of HFRS through impacts on hantavirus reservoirs. Previous studies have shown a clear association between climate change and the risk of HFRS [9-16], and researchers have suggested that this association is based on direct and indirect effects of climate change. Climate change and the associated warming of global temperatures contribute to abnormal climate patterns including unusually hot summers and cold winters with heavy snowfall. Air pollution is thought to be one of the factors that contribute to global warming. Therefore, air pollution could affect the occurrence of HFRS, because air pollution is associated with changes in climate variables. Furthermore, it can be postulated that worsening air pollution could lead directly to an increase in the occurrence of HFRS, because air particles are a major mediator of hantavirus transmission. However, to date, a correlation between air pollution and rodent transmitted infectious diseases, including HFRS, has not been demonstrated.

In the present study, we aimed to verify this hypothesis using data on particulate matter smaller than 10 μm (PM_10_) to represent air pollution. We also included climate variables in our analyses to assess the independent correlation between PM_10_ and HFRS under the consideration for climate.

## Methods

### Data collection

This ecological correlation study was approved by the institutional review board at the Seoul National University Hospital (no. H-1203-066-402) and was carried out at the Seoul National University College of Medicine (Seoul, South Korea) from January 2012 to June 2012. Since 1976, HFRS has been legally designated as a nationally notifiable disease in South Korea. Therefore, once a new HFRS case is diagnosed, doctors or other agents are required to report the occurrence of the disease according to the standard form determined by the Korea Centers for Disease Control and Prevention (KCDC). After notification, the occurrence of HFRS is reported every month on the KCDC web site. HFRS is diagnosed using both clinical criteria and positive test results for hantavirus, hantavirus antigen, or hantavirus RNA sequences in blood or tissue. South Korea is divided into 16 provinces (15 land based provinces and one island, Jeju province). We obtained monthly HFRS case data for the years of 2001-2010 from a database compiled by the KCDC; but data from Jeju province was excluded as only one HFRS case was reported.

Particulate matter (PM) was used as an index of air pollution in the current study. PM is a mixture of solid particles and liquid droplets that vary in size, and has been widely used for the investigation of health effects of air pollution [17]. Among several size categories, PM_10_ has been monitored in South Korea. Data on the concentrations of PM_10_ (μg/m^3^) between 2001 and 2010 were obtained from the national database of the Ministry of Environment.

**Figure 1:**
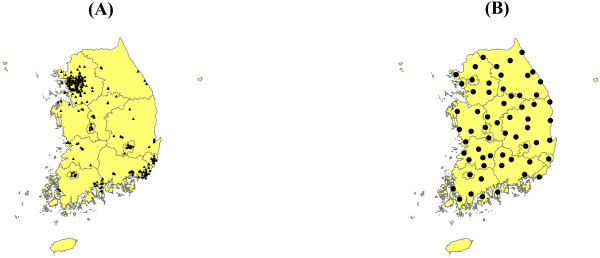
**Location of monitoring stations in South Korea.** (**A**) air pollution stations; (**B**) weather stations. The stations located in Jeju province (island at the southern end) are not used in the present study.

When assessing the correlation between air pollution and HFRS, climate variables may act as a confounding factor. Therefore, we also collected climate data for the years 2001 to 2010. Monthly climate data, including mean, maximum, and minimum land surface air temperatures on the Celsius scale, relative humidity (%), and cumulative precipitation (mm/1000), were obtained from the national database of the Korea Meteorological Administration. In our study, 233 air pollution stations were used to evaluate PM_10_ concentrations and 62 weather stations were used to evaluate the climate variables. These stations have been monitoring the PM_10_ concentrations and the climate data to obtain representative data for South Korea (Figure 1).

### Statistical analysis

All analyses and calculations were performed using SAS version 9.2 (SAS Institute Inc., Cary, NC, USA). A Poisson regression model of the monthly HFRS occurrence rate against air pollution measures and other confounders was fitted to obtain estimates of the relative risk of HFRS occurrence associated with the air pollution. We incorporated time lags of 1 to 6 months for the climate variables in the model. For PM_10_, time lags extending up to 12 months were also considered to verify the lag effect of PM_10_. A multivariate analysis with forward selection was used to avoid multicollinearity and to find a good model for describing the observed number of HFRS cases. For forward selection, the following criteria were considered: statistical significance (*P* value), goodness of fit (deviance), and a clinical interpretation. From the mean, maximum, and minimum temperatures, the most statistically significant variable was selected for the multivariate model. The Durbin-Watson statistic was applied to detect the presence of autocorrelations between HFRS variables. The relative goodness of fit in the multivariate model based on climate variables, seasonality, and PM_10_ was compared with the model that did not include PM_10_ using the scores of the Akaike information criterion. Finally, HFRS cases for 2011 were predicted using the multivariate models driven by the database for 2001–2010. Values of *P* <0.05 were considered to be statistically significant.

## Results

### Temporal dynamics of HFRS cases according to the PM_10_ concentration

**Figure 2:**
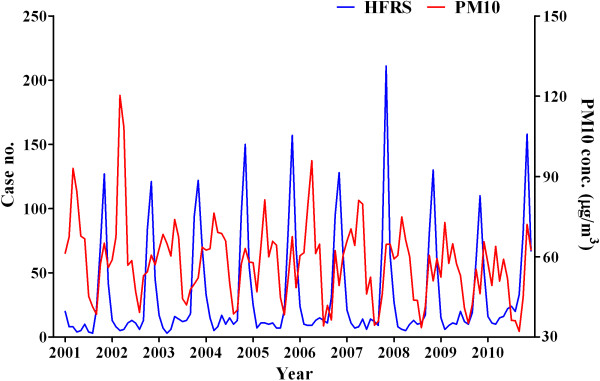
Temporal dynamics of HFRS cases and PM10 concentrations between 2001 and 2010.

A total of 3952 HFRS cases were identified from 2001 to 2010. Figure 2 shows the temporal dynamics of HFRS cases and PM_10_ concentrations. The results showed that cases of HFRS reach a peak in autumn. There was no significant autocorrelation between monthly HFRS occurrences (*P* > 0.05 determined by the Durbin-Watson statistic). In the univariate analyses, the associations between HFRS and PM_10_ were significant, although the direction of the correlations changed according to the different time lags (Table 1). For every 1.0 μg/m^3^ increase in the previous 3-month PM_10_ level, the monthly cases of HFRS decreased by 6.7%. In contrast, every 1.0 μg/m^3^ increase in the previous 7-month PM_10_ level corresponded to an increase of 3.6% in the number of monthly HFRS cases. The PM_10_ effects were different in the multivariate analyses of adjusted climate variables with the correlation directions and significances changing for PM_10_ without a time lag and for PM_10_ with some time lags.

**Table 1:** Poisson regression model between the air pollutant and hemorrhagic fever with renal syndrome

**Time lag**	**Univariate**		Multivariate^*^	
	**RR (95% CI)**	***P***	**RR (95% CI)**	***P***
No time lag	0.998 (0.995–1.000)	.025	1.013 (1.008–1.017)	< .001
1–month lag	0.972 (0.970–0.975)	< .001	1.001 (0.997–1.004)	.785
2–month lag	0.938 (0.935–0.940)	< .001	0.991 (0.987–0.995)	< .001
3–month lag	0.933 (0.931–0.936)	< .001	0.983 (0.979–0.987)	< .001
4–month lag	0.964 (0.961–0.966)	< .001	0.992 (0.988–0.996)	< .001
5–month lag	0.997 (0.995–1.000)	.022	0.991 (0.988–0.995)	< .001
6–month lag	1.024 (1.022–1.026)	< .001	1.005 (1.002–1.008)	.001
7–month lag	1.036 (1.034–1.037)	< .001	1.006 (1.004–1.009)	< .001
8–month lag	1.031 (1.030–1.033)	< .001	1.002 (1.000–1.005)	.036
9–month lag	1.016 (1.014–1.018)	< .001	0.999 (1.012–1.031)	.360
10–month lag	1.005 (1.002–1.007)	< .001	0.993 (0.989–0.997)	.001
11–month lag	1.000 (0.998–1.002)	.827	0.997 (0.993–1.002)	.208
12–month lag	0.990 (0.988–0.992)	< .001	0.999 (0.995–1.003)	.572

*CI* confidence interval, *RR* relative risk.

Dependent variable is the occurrence of hemorrhagic fever with renal syndrome.

^*^Adjusted for seasonality and climate variables.

### PM_10_-based forecasting model for HFRS after adjusting for climate variables and seasonality

**Figure 3:**
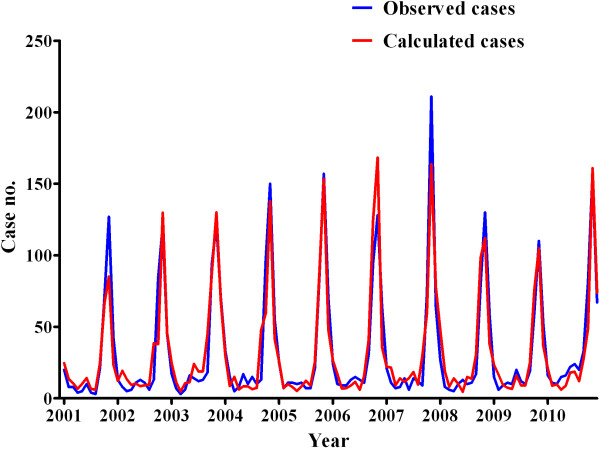
Matching of the calculated number of HFRS cases to the observed number of HFRS cases from 2001 to 2010.

**Figure 4:**
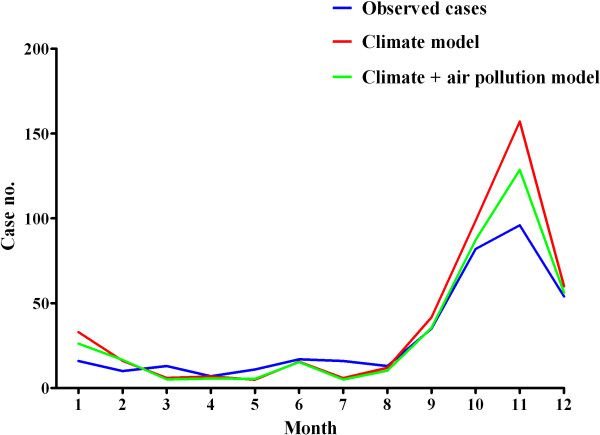
**Number of HFRS cases in 2011 predicted using the climate model and the combined climate and air pollution model.** Blue line, observed cases; red line, climate model; Green line, climate + air pollution model.

The models predicting the number of HFRS cases were evaluated using multivariate Poisson regression analyses with forward selection (Table 2). For the model, a humidity variable with a 4-month lag and a precipitation variable with a 3-month lag were selected. The mean temperature variable was a more significant predictor of HFRS than the maximum and minimum temperature variables. Therefore, the mean temperature with a 1-month lag was selected for the HFRS model. When considering the PM_10_ and the climate variables, the PM_10_ variable without a time lag was selected in the final model using forward selection steps. For every 1.0 μg/m^3^ increase in the PM_10_ level, the number of monthly cases of HFRS increased by 0.013. The Akaike information criterion scores for in the climate model and the combined climate and air pollution model were 1142.9 and 1109.5, respectively. Figure 3 shows the lines corresponding to the fitted and observed HFRS cases using the final models from 2001 to 2010. The calculated number of HFRS cases matched well with the observed number of HFRS cases. We then predicted the number of HFRS cases in 2011 based on the final models and compared this value with the observed number of HFRS cases (Figure 4).

**Table 2:** Multivariate Poisson regression model for hemorrhagic fever with renal syndrome

	**Climate model**			**Climate + air pollution model**		
		**RR (95% CI)**	***P***		**RR (95% CI)**	***P***
Seasonality	Winter	1 (Reference)		Winter	1 (Reference)	
	Spring	0.998 (0.853–1.168)	.981	Spring	0.813 (0.683–0.967)	.019
	Summer	1.275 (1.062–1.529)	.009	Summer	1.146 (0.952–1.380)	.150
	Autumn	1.818 (1.562–2.116)	< .001	Autumn	1.656 (1.419–1.933)	< .001
Humidity	4–month lag	1.102 (1.094–1.110)	< .001	4–month lag	1.102 (1.094–1.110)	< .001
Precipitation	3–month lag	1.022 (1.018–1.026)	< .001	3–month lag	1.018 (1.014–1.022)	< .001
Mean temperature	1–month lag	1.022 (1.013–1.032)	< .001	1–month lag	1.038 (1.027–1.049)	< .001
PM_10_				No time lag	1.013 (1.008–1.017)	< .001

*CI* confidence interval, *RR* relative risk, *PM*_*10*_ particulate matter smaller than 10 μm.

Dependent variable is the occurrence of hemorrhagic fever with renal syndrome.

## Discussion

HFRS is a critical infectious disease having a 15% fatality rate [18]. However, the annual incidence of HFRS has not decreased despite having received considerable interest. In this study, we used three datasets, including data on HFRS, climate, and air pollution, and attempted to demonstrate the correlation between HFRS and air pollution when climate variables were considered. PM_10_ levels were positively or negatively associated the occurrence of HFRS according to the time lags. These correlations were affected or not affected by climate variables. However, the strongest association between HFRS and PM_10_ was observed when using no time lags. This is the first report to document an association between HFRS and PM_10_ level.

The activity of rodents plays a primary role in the transmission of HFRS because HFRS is mainly transmitted via aerosols of hantavirus-containing excreta from rodents. Previous studies have revealed that climate change is associated with the occurrence of HFRS. The association between climate change and HFRS can be explained by a number of mechanisms. Climate variables including temperature directly affect the activity of rodents [19]. Furthermore, climate change potentially constitutes an indirect link to the density of rodents via changes in food resources [20]. There is recent evidence that the degree of viral infectivity or replication rate may be controlled by climate variables [21,22]. It can be assumed that climate variables also affect human activity and, subsequently, human contact with rodent excreta changes. However, the burden of HFRS occurrence cannot be explained by climate change alone. The current study focused on the effect of air pollution, because air pollution is closely related to climate change [23].

We found air pollution had a significant association with the number of HFRS cases in the univariate analysis. However, this association was complex in that the correlation direction changed according to the time lags. The multivariate analysis showed that the correlation directions and significances of the air pollution effect with time lags were different after adjustment of the climate variables. PM_10_ values with some time lags affected HFRS risks irrespectively of climate variables. However, PM_10_ without a time lag was selected for the best fit model and had a direct effect on HFRS cases without reference to climate variables. This best fit model can be explained by the route of HFRS transmission. As an index of air pollution, we assessed PM_10_, which is composed of fine particles suspended in a gas or liquid. Therefore, PM_10_ can serve as a transmission indicator for hantavirus. The body of evidence strongly suggests a link between air pollution and respiratory infection. This observation is explained primarily by the modulation of the immune system [24]. Likewise, air pollution may affect the frequency of HFRS cases by changing the viral infectivity and immunity of both humans and rodents [25,26]. However, these potential mechanisms have been primarily explored in the field of respiratory infection, and to date there has been no significant advance in understanding the processes involved.

There are limitations that need to be noted. Other climate factors which were not considered (e.g.; wind and solar radiation) or climate variables beyond a 6-month time lag might also influence correlations. Furthermore, we did not add information on the dynamics of rodent population. The virus is labile for remaining infective if there is not a sustained natural source close in time from HFRS cases. This issue should be examined in future studies attempting to drive the mechanisms linking air pollution and HFRS.

## Conclusions

Results of previous studies have demonstrated that air pollution can change the epidemiology of respiratory infectious diseases. However, this hypothesis has not yet been widely investigated, especially in the field of vector-borne diseases. Here we present, for the first time, an association between HFRS cases and PM_10_ as an index of air pollution. Building on the present study as a starting point, further studies are needed to address the effects of air pollution on other types of infectious diseases.

## Competing interest

The authors declare that they have no conflicts of interest.

## Authors’ contributions

SSH collected the data, performed the study, interpreted the data, and wrote the manuscript. SK (Sunhee Kim) and YC preformed statistical analyses. SK (Suhnggwon Kim) designed the study and advised on the results. YSK designed the study, analyzed the results, wrote the manuscript, and edited the manuscript. All authors read and approved the final manuscript.

## Pre-publication history

The pre-publication history for this paper can be accessed here:

http://www.biomedcentral.com/1471-2458/13/347/prepub
